# Endothelial cell dysfunction: Implications for the pathogenesis of peripheral artery disease

**DOI:** 10.3389/fcvm.2022.1054576

**Published:** 2022-11-16

**Authors:** Mary M. Kavurma, Christina Bursill, Christopher P. Stanley, Freda Passam, Siân P. Cartland, Sanjay Patel, Jacky Loa, Gemma A. Figtree, Jonathan Golledge, Sarah Aitken, David A. Robinson

**Affiliations:** ^1^Heart Research Institute, The University of Sydney, Sydney, NSW, Australia; ^2^South Australian Health and Medical Research Institute, Adelaide, SA, Australia; ^3^Faculty of Health and Medical Science, University of Adelaide, Adelaide, SA, Australia; ^4^Central Clinical School, Faculty of Health and Medicine, The University of Sydney, Sydney, NSW, Australia; ^5^Royal Prince Alfred Hospital, Sydney, NSW, Australia; ^6^Faculty of Health and Medicine, The University of Sydney, Sydney, NSW, Australia; ^7^Kolling Institute of Medical Research, Royal North Shore Hospital, Sydney, NSW, Australia; ^8^Queensland Research Centre for Peripheral Vascular Disease, College of Medicine and Dentistry, James Cook University, Townsville, QLD, Australia; ^9^The Department of Vascular and Endovascular Surgery, Townsville University Hospital, Townsville, QLD, Australia; ^10^Concord Institute of Academic Surgery, Concord Hospital, Sydney, NSW, Australia

**Keywords:** peripheral artery disease (PAD), inflammation, therapeutics, endothelial cell dysfunction, thrombosis, angiogenesis, vascular tone

## Abstract

Peripheral artery disease (PAD) is caused by occluded or narrowed arteries that reduce blood flow to the lower limbs. The treatment focuses on lifestyle changes, management of modifiable risk factors and vascular surgery. In this review we focus on how Endothelial Cell (EC) dysfunction contributes to PAD pathophysiology and describe the largely untapped potential of correcting endothelial dysfunction. Moreover, we describe current treatments and clinical trials which improve EC dysfunction and offer insights into where future research efforts could be made. Endothelial dysfunction could represent a target for PAD therapy.

## Introduction

Peripheral artery disease (PAD) is defined by the partial or total blockage of the arteries supplying the lower extremities. More than 200 million people world-wide are affected with >6.5 million people living with PAD in the United Stated of America (USA) alone (1). PAD has significant impact because of the frequent need for medical and surgical treatment. Using 2014 data from National Inpatient Sample, Kohn and colleagues recently identified that the cost burden of hospitalization for PAD patients in the USA was ~$6.3 billion per year (2). This medical and economic cost will rise; PAD prevalence is increasing due to the obesity and diabetes pandemic. People presenting with PAD have a higher risk of all-cause and cardiovascular mortality than those presenting with risk in coronary artery diseases (CAD) (3), but PAD has received limited attention in the development of treatments.

PAD may be classified into 3 clinical presentations—asymptomatic or atypical, intermittent claudication, and chronic limb-threatening ischemia (CLTI) (4). Most patients, ~20–50%, are asymptomatic or display atypical symptoms whereas 10–30% of patients display typical features of intermittent claudication i.e., exertional leg pain in ≥1 muscle group(s), relieved by rest (5). CLTI is the severe stage of PAD, including ischemic foot pain at rest, non-healing wounds/ulcerations and in severe cases, gangrene due to arterial insufficiency, necessitating revascularization surgery or amputation. The presentation of PAD may not reflect the severity of limb ischemia and because of the varying presentations, many patients are misdiagnosed or underdiagnosed.

PAD is generally viewed as a large vessel atherosclerotic disease but studies report that its pathophysiology differs from atherosclerosis in other vessel beds. Narula and colleagues recently summarized histological differences of plaques in patients with PAD and CAD (6). The authors suggested that the blockage of the coronary vessels in acute coronary syndromes was principally caused by luminal thrombus, with 65–75% suggested to be due to plaque rupture and 25–35% to plaque erosion (defined as the presence of luminal thrombosis in the absence of plaque rupture). In contrast, ~66% of peripheral arteries from CLTI were occluded by thrombosis in the absence of significant atherosclerosis, defined as normal intima or adaptive intimal thickening (6). The same authors also identified blood clots in the small distal arteries, proposing that local changes in these vessels could precipitate thromboembolic events, however it is unclear if these events were due to the process of amputation or if they occurred prior. The microvasculature also plays a significant role in PAD pathophysiology since microvascular dysfunction can increase amputation risk (7). These studies suggest that our knowledge in PAD pathophysiology is limited. Endothelial cell (EC) heterogeneity and plasticity has been confirmed in different organs and vascular beds in peripheral arteries (8–12), suggesting that EC dysfunction may represent a spectrum of EC phenotypes (13). This isn't surprising since the pattern and behavior of ECs are shaped by their environment and the tissues they reside in. Given ECs line the entire vascular tree, the study of EC function and dysfunction in peripheral arteries could be key to understanding some of these differences. Certainly, a healthy endothelium not only acts as a barrier between blood and surrounding tissues, but is considered an endocrine organ, regulating exchanges between the blood stream and tissues to control constriction and dilation and maintain vessel tone. The endothelium also inhibits thrombosis, reduces leukocyte adhesion and transmigration, limits atherogenesis, and is responsible for the formation of new blood vessels necessary for repair during damage.

In this review, we summarize EC function(s) that are altered in PAD. We highlight current therapeutics and treatments being investigated in clinical trials that impact EC function(s) as well as offer insight into where future research efforts could be made. Knowledge of how EC function and dysfunction contributes to PAD pathophysiology could have significant implications for therapeutic and diagnostic approaches for this disease.

## EC dysfunction in pad

### Inflammation

In homeostasis, leukocytes move in and out of the vascular system and tissues and are in constant surveillance of their microenvironment waiting for a signal. In response to stimuli, these cells are recruited to inflamed tissues, where they “clean up” the injury and contribute to repair. In atherosclerosis, damage to the endothelium (e.g., increased turbulence of blood flow, high blood pressure, high cholesterol, high glucose, oxidation etc.,) can upregulate multiple mediators governing leukocyte recruitment. Chemokines, cytokines, and other inflammatory mediators regulate the expression of adhesion molecules on both the endothelium, neutrophil, and monocyte surface to influence the three-step process of leukocyte recruitment: rolling, activation, and adhesion on the endothelium which involves E-, L- and P-selectins. While rolling is essential for leukocyte adherence, it does not necessarily lead to firm adhesion; firm adhesion requires activation of integrins (by selectin or chemokine engagement) and their interaction with ICAM-1 and VCAM-1 (intercellular adhesion molecule-1 and vascular cell adhesion molecule-1), which results in the complete arrest of the leukocyte. Leukocytes then transmigrate between the endothelium into the interstitial space toward a chemotactic stimulus, such as, CC-chemokine ligand-2 (CCL-2) (14).

Some of these cellular interactions have been implicated in PAD. For example, circulating levels of monocytes are significantly and independently associated with PAD (15), with high neutrophil-lymphocyte ratio a potential predictor of PAD severity (16, 17). Circulating chemokines and inflammatory markers expressed by leukocytes including high sensitivity C-reactive protein (hs-CRP), interleukins (IL), and matrix metalloproteinases (MMPs) are also upregulated in PAD patients, and in some cases associate with severity of disease (18–21). Indeed, a systematic review and meta-analysis of 47 studies involving >21,000 PAD patients, identified high levels of hs-CRP to predict the risk of major adverse cardiovascular events and mortality (22). Platelets release low abundance, highly active molecules including chemokines/chemokine ligands and angiogenic factors including chemokine ligand-5 (CCL-5) and platelet-derived growth factor (PDGF), which support leukocyte-platelet interaction and migration of neutrophils and monocytes to the developing atherosclerotic site (23). Indeed, Barrett et al., recently identified that platelets induced the migration of monocytes into atherosclerotic lesions of *Ldlr*^−/−^ mice *via* upregulation of monocyte suppressor of cytokine signaling 3 (SOCS3) (24). Thus, platelets also contribute to inflammation, and with increased inflammatory burden, there is an increase in PAD prevalence (25). Another cytokine important for migration of leukocytes into the vessel wall is CCL-2. CCL-2 infusion of the femoral artery of rabbits following hindlimb ischemia increased monocyte accumulation in the vessel wall (26); a finding that was inhibited with ICAM-1 monoclonal antibody treatment (27), suggesting that monocyte adhesion to the endothelium in ischemia involves CCL-2 and ICAM-1. Importantly, circulating CCL-2 levels are increased in PAD (18, 28), associating with increased CCL-2 protein expression and macrophage content in limb tissues from patients (29).

**Figure 1:**
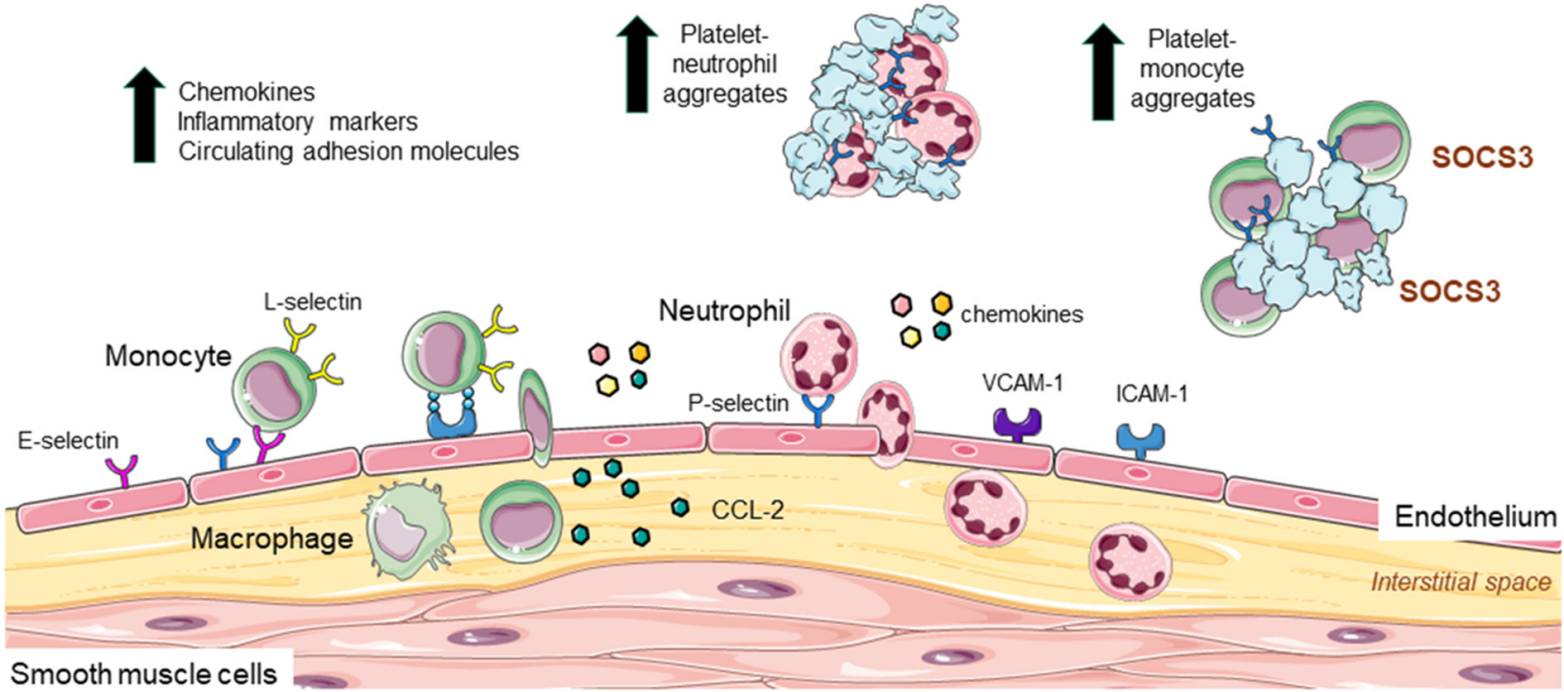
Leukocyte and platelet adhesion, recruitment to the endothelium, and contribution to inflammation in PAD. In atherosclerosis increased inflammation upregulates the expression of VCAM-1 and ICAM-1 as well as E- and P- selectin on endothelial cells. These adhesion markers are involved in the process of leukocyte recruitment (rolling, activation, and adhesion to the endothelium), with L-selectin also implicated in adhesive interactions between leukocytes and endothelial cells. Once adhered, leukocytes migrate into the interstitial space toward a chemotactic mediator such as CCL-2. Here, monocytes differentiate into macrophages. Monocytes are independently associated with PAD and increased neutrophil: lymphocyte ratio is linked to PAD severity. Circulating chemokines, inflammatory markers (e.g., CRP, interleukins, and matrix metalloproteinases), and adhesion molecules (ICAM and P-selectin) are up regulated in PAD and associated with disease severity. Platelets also secrete molecules that support platelet-neutrophil and platelet-monocyte interaction and adhesion. Circulating platelet-leukocyte interactions are considered a biomarker for PAD.

Additional reports of the role of cell adhesion molecules come from the Edinburgh Artery Study, which described increased soluble levels of ICAM-1 associating with PAD diagnosis (30). In another study, soluble VCAM-1 levels were associated with worse PAD prognosis (31). From the selectins: E-selectin is EC specific whereas P-selectin is expressed by both ECs and platelets. Higher levels of soluble E-selectin have been reported in PAD patients, particularly in diabetes, and reflecting endothelial activation (32). Circulating P-selectin levels are also associated with PAD severity (33–35) and in the Multi-Ethnic Study of Atherosclerosis, a prospective large cohort study involving >6,800 participants, P-selectin levels were significantly associated with lower ankle-brachial index ratios as well as PAD prevalence (36). P-selectin's involvement in leukocyte adhesion was confirmed *in vitro*; human recombinant P-selectin increased neutrophil adhesion to platelets in the presence of plasma from healthy individuals (34), suggesting that neutrophil adhesion could also occur with activated endothelium expressing P-selectin in PAD patients. Moreover, a link between platelets and SOCS3-mediated activation in PAD was observed, with the SOCS1:SOCS3 ratio negatively correlating with IL-1β, but also with monocyte-platelet aggregates, P-selectin and CD40 (24). Indeed, increased circulating leukocyte-platelet aggregates are proposed as a biomarker of PAD severity (17). An in-depth summary of inflammatory biomarkers in PAD was recently reviewed (37). Figure 1 summarizes the endothelial, leukocyte and platelet contribution to inflammation in PAD.

### Platelet activation and thrombosis

Under normal circumstances, the endothelium exquisitely controls endothelial-platelet interactions and the balance between coagulation and anticoagulation in the vessel wall. ECs generate nitric oxide (NO) and prostacyclin, molecules which directly inhibit platelet activation. They express tissue factor pathway inhibitor, a potent anticoagulant, which limits tissue factor-inducible activation of factor VII and X (38). ECs also express co-factors for antithrombin III, or synthesize thrombomodulin (a thrombin receptor), which can directly reduce plasma thrombin levels upon thrombin binding, an effect that can increase the activity of the anticoagulant, protein C (39). Furthermore, ECs secrete tissue-type and urokinase-type plasminogen activator (t-PA and u-PA, respectively) to activate fibrinolysis and fibrin degradation. Because thrombosis is strongly implicated in PAD (6), the EC-platelet interaction may be more central to PAD pathogenesis. Indeed, PAD patients have elevated levels of circulating platelets (40) and increased platelet aggregability (41), with mean platelet volume increasing with PAD severity (42). Circulating tissue factor (43), t-PA (40) and factors IX and XI (44) are also increased, whereas tissue factor pathway inhibitor (43) and protein C levels are decreased (44); the latter associating with endothelial injury (44). Elevated von Willebrand factor and fibrinogen levels are also independently associated with the risk of development of PAD (45), with increased fibrinogen and D-dimer levels predictive of increased risk of mortality in PAD patients (22). Soluble thrombomodulin levels are increased in symptomatic PAD vs. asymptomatic age-matched control subjects (46), which is significant since elevated levels may reflect EC dysfunction in PAD (47, 48).

Interestingly, medial calcification and calcified nodules were identified in 70% of CLTI peripheral arteries examined (6, 49). Calcified nodules, accompanied by fibrin could pierce or disrupt the fibrous cap causing EC loss and plaque rupture (50). This is important since vascular calcification may predict poorer outcomes in PAD (51). However, reports suggest that calcification may also stabilize plaque (52). The role of calcification in PAD is not well established and requires further study.

### Vessel tone

To meet physiological demand and manage blood flow, the endothelium dilates arteries by relaxing the underlying smooth muscle *via* a range of mechanisms. The most well studied is NO. Under normal physiological conditions, the formation of NO is dependent on calcium facilitated calmodulin binding to homodimeric endothelial nitric oxide synthase (eNOS). Bound calmodulin then facilitates, 6R-tetrahydrobiopterin (BH4)-dependent electron transfer across eNOS to catalyze the conversion of L-arginine to NO and L-citrulline. However, roles for cyclooxygenase (COX)-derived prostoglandins (53), membrane hyperpolarization (54), epoxyeicosatrienoic acids (55), myoendothelial gap junctions (56) and oxidants (57) can also control arterial tone in an endothelial-dependent manner. The specific mechanism of regulation differs depending on how the artery is stimulated, sex, and the location of the artery in the circulatory system. However, arguably the biggest variable that dictates which mechanism is employed is the health state of the artery.

In PAD patients many of the above mechanisms become dysfunctional. For instance, flow mediated dilation (FMD), a surrogate for endothelial function, and commonly associated with endothelial production of NO, is reduced (58). Consistent with this, PAD patient plasma and urine showed decreased BH4, cyclic guanosine monophosphate (cGMP) and decreased NOX's, pointing toward a decrease in endothelial NO bioavailability (59). Ismaeel et al. (60) has since shown a range of increased oxidative stress markers in PAD, proposing these as mechanisms by which NO bioavailability is lost, and is somewhat in agreement with other studies. For example, administration of L-arginine to patients to stimulate NO production, or oxypurinol to decrease oxidative stress (and thus increase NO bioavailability) increased FMD, restored blood flow and decreased patient symptoms (61, 62). However, despite the promise of these short-term studies, longer term clinical trials over 6 months in the NO-PAIN study showed that oral administration of L-arginine did not improve FMD or improve any NO biochemical parameters in patients with intermittent claudication (63). An additional strategy to increase NO bioavailability is to modulate the eNOS enzyme directly. In this regard the β-adrenergic receptors (βARs) may have therapeutic potential, particularly the β_3_AR isoform. β_3_AR agonists stimulate vasodilation *via* their ability to modulate eNOS activity and NO production (64, 65). Moreover, we showed that activation of β_3_AR restored NO and the redox balance, improving vasodilation and EC function in a mouse model of diabetic PAD (66).

**Figure 2:**
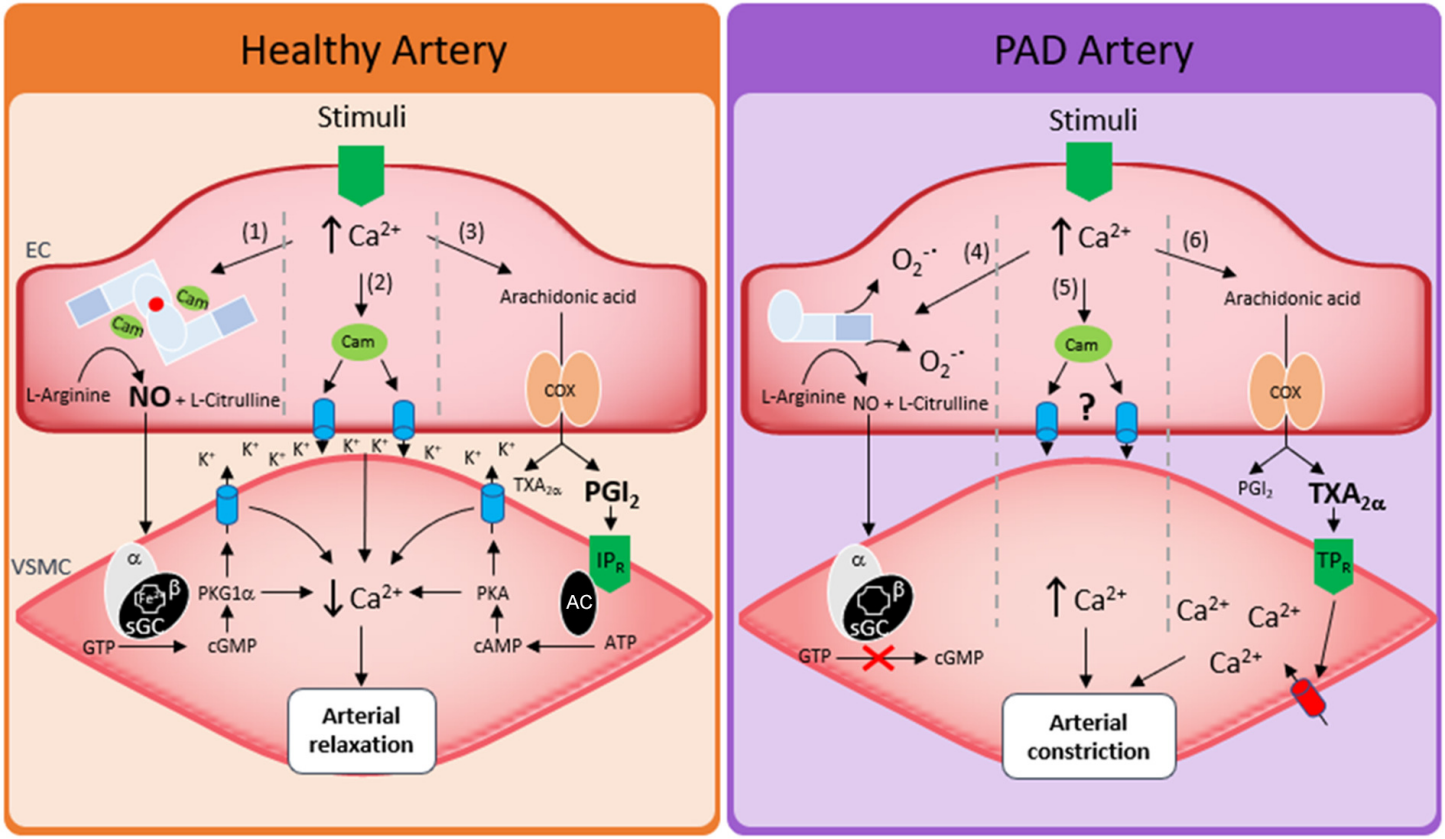
Regulation of vessel tone in healthy arteries and in PAD. Left hand side shows three mechanisms of arterial relaxation in healthy arteries. (1) Nitric oxide induced arterial relaxation, endothelial calcium binds calmodulin which in turn facilitates eNOS mediated oxidation of L-Arginine forming NO and citrulline. NO then diffuses from the endothelial cell (EC) to the vascular smooth muscle cell (VSMC) where it activates soluble guanylate cyclase (sGC). sGC then converts guanosine triphosphate (GTP) into cyclic guanosine monophosphate (cGMP). cGMP activates protein kinase G1a (PKG1α) which lowers smooth muscle calcium through potassium channel mediated hyperpolarization of smooth muscle cells. (2) Increased endothelial calcium binds calmodulin which in turn activates small and intermediate conductance calcium activated potassium channels. Potassium efflux through these channels hyperpolarizes vascular smooth muscle *via* direct spreading of membrane potential or *via* activation of smooth muscle ion channels. (3) Endothelial calcium increases and increases arachidonic acid formation from membrane phospholipids. Arachidonic acid is the metabolized *via* cyclooxygenase 1 or 2 (COX) to form prostaglandins. The dominant prostaglandin formed is prostacyclin (PGI_2_). PGI_2_ then activates the IP receptor (IPR) which converts adenosine triphosphate (ATP) into cyclic adenosine monophosphate (cAMP) in an adenylyl cyclase (AC)-dependent manner, cAMP then activates protein kinase A (PKA) which lowers smooth muscle calcium through potassium channel mediated hyperpolarization of smooth muscle cells. Right hand side shows how peripheral artery disease affects arterial relaxation. (4) Increased endothelial calcium increases production of superoxide (O_2_^−•^) *via* stimulation of uncoupled eNOS or stimulation of other oxidant sources such as NADPH oxidases (NOX's). Any NO that has been produced may be ineffective as its target sGC has been oxidized or become heme free. (5) Mechanisms of potassium channel induced hyperpolarization have not been investigated. (6) arachidonic acid breakdown results in increased thromboxane A2 (TXA2α) or increased activation of thromboxane receptor (TPR) activation.

The impact of PAD on other vasodilatory pathways is less investigated but should not be overlooked. Evidence already exists of other pathways such as the COX-dependent regulation of vascular tone that may be disrupted in PAD models (67), and that selective inhibition of COX-2 offers clinical improvement in intermittent claudication (68). Given that the contribution to vascular tone of NO decreases as vessel size decreases (69), future research should also investigate the effects of PAD on non-NO mechanisms of arterial dilation. A summary of these pathways is described in Figure 2.

### Angiogenesis

In hemostasis, injury to blood vessels (e.g., ischemia) activate ECs, which then sprout, migrate, proliferate, and form EC tubules, driven by hypoxia-induced mediators, the most characterized being vascular endothelial growth factor (VEGF). Interestingly, in clinical practice and in animal models of cardiovascular disease, the endogenous angiogenic responses are impaired, particularly with aging (70, 71), in diabetes (72–74) or in dyslipidemia (71, 75, 76). Thus, stimulating angiogenesis in local ischemic tissues could be beneficial in PAD and other vascular diseases. However, to date, all large clinical trials delivering angiogenic factors, including VEGF to people suffering from ischemic diseases such as PAD have shown little benefit (77). Furthermore, the role of angiogenesis in atherosclerosis is conflicting. For example, in a rabbit carotid artery collar model of intimal hyperplasia, adenoviral delivery of VEGF-A, -B, -C and -D increased intimal thickening which was positively correlated with neovascularisation (78). VEGF and other angiogenic molecules including fibroblast growth factor (FGF) were shown to accelerate atherosclerosis in animal models (79, 80), whereas anti-angiogenic therapies reduced atherosclerosis development (81). In contrast, systemic inhibition of the VEGF receptor attenuated established atherosclerosis in high fat diet-fed *Apoe*^−/−^ mice (82). Interestingly, plasma concentrations of VEGF-A, but not VEGF receptor-1, are significantly elevated in PAD patients vs. healthy controls (83). In support of this, a significant increase in plasma VEGF was observed in patients with intermittent claudication vs. CLTI, suggesting that VEGF may act as a biomarker or causal factor in disease (83). Indeed, Stehr et al. found increased VEGF levels associated with increased PAD severity (84). What is clear from these studies is that our knowledge of the complex angiogenic pathways and responses to ischemia in PAD is limited.

Other molecules of interest that can promote angiogenesis include TNF-related apoptosis-inducing ligand (TRAIL) and β_3_AR. TRAIL is a protein discovered for its ability to selectively kill tumor cells but leave normal cells resistant to its cytotoxic actions (85, 86). Interestingly, in ischemic cardiovascular diseases including PAD, TRAIL levels in the circulation are suppressed (87–90) and in the cardiovascular system TRAIL appears to have homeostatic rather than cytotoxic properties. For example, the presence of TRAIL attenuated atherosclerosis in mice (87, 91), in part by resolving inflammation and improving macrophage function (87), reducing oxidative-stress-induced EC dysfunction (92), and increasing eNOS activity to stimulate intracellular NO production in ECs (93). In the latter study, TRAIL stimulated NO production *via* a NOX-4-dependent mechanism (93). Furthermore, we and others showed that exogenous TRAIL treatment stimulated *in vitro* EC processes of angiogenesis (proliferation, migration and tubule formation) (93–95) and promoted stable collateral vessels in the ischemic limb of mice (93). Activation of the β_3_AR can also stimulate processes of angiogenesis. For example, the specific β_3_AR agonist BRL37344 increased retinal EC proliferation and migration *in vitro*, whereas stimulation of the β1 isoform, β_1_AR had no effect (96). Furthermore, nebivolol-induced angiogenesis in a mouse model of aortic sprouting was abrogated with β_3_AR deletion, demonstrating the importance of β_3_AR in neoangiogenesis (97). More recently, we showed that administration of CL316,243, a specific β_3_AR agonist, stimulated human umbilical vein EC migration and tubule formation in a NOS-dependent manner *in vitro*, and crucially, CL316,243 improved blood perfusion and angiogenesis in a mouse model of diabetic PAD (98). These findings support TRAIL and β_3_AR agonist modulators as promising new therapeutic agents for the treatment of PAD.

## Current treatments and clinical trials which target and improve EC function

Current gold-standard treatment of PAD includes the management of symptoms such as exercise therapy, medical therapy (e.g., lipid control, blood pressure control, anti-platelet therapy and diabetes control), and revascularization to reduce the risk of myocardial infarction (MI) and stroke. Interventions for PAD are guided by disease stage; guidelines for management have been published by the European Society of Vascular Surgery and European Society of Cardiology in 2018 (99), the AHA and American College of Cardiology (ACC), 2016 (100), the Global Vascular Guidelines in 2019 (4) and the Asia-Pacific Consensus Statement on PAD Project Committee (APPADC) (101). These guidelines are summarized in Table 1. Below, we focus our attention on some of these therapies as well as describe emerging therapies known to impact EC functions.

**Table 1:** Summary of Class I PAD treatment guidelines.

**Recommendation**	**Level of evidence**			
	**AHA/ACC** **2016**	**ESC/ESVC** **2018**	**GVG 2019**	**APPACD** **2021**
Lipid lowering drugs for all PAD patients	A	A	A	A
Antihypertensive therapy exclusively for hypertensive PAD patients to reduce chance of MI, stroke, heart failure or cardiovascular death	A	B	B	A
Diabetes therapies to maintain	C	C	B	B
Smoking cessation for PAD patients who smoke cigarettes or other forms of tobacco	A	B	A	A
Antiplatelet therapy to reduce risk of MI or stroke	A	C	A	A
Antithrombotic therapy to reduce risk of MI or stroke	A	A	A	A
Exercise therapy to improve claudication symptoms	A	C	N/A	A
Annual influenza shot	C	N/A	N/A	C
Healthy diet	N/A	C	N/A	N/A
Endovascular treatment for claudication	N/A	C	B	B

AHA/ACC, American Heart association and American College of Cardiology; ESC/ESVS, European Society of Vascular Surgery and European Society of Cardiology; GVG, Global Vascular Guidelines; APSAVD, Asia-Pacific Society of Atherosclerosis and Vascular Disease. A, data derived from multiple randomized control trials or meta data; B, data derived from a single randomized clinical trial or large non-randomized clinical trials; C, consensus of expert opinion and/or small studies, retrospective studies and registries; N/A, not applicable.

### Exercise

Exercise training has positive effects on endothelial function. This is particularly evident in CAD where exercise training improved endothelium-dependent vasodilation that resulted in a 4-fold phosphorylation of eNOS^1,177^ from isolated arteries 4 weeks later (102). Increased endothelial function after exercise is also observed in patients with hypertension (103), and in type-1 and type-2 diabetes (104, 105). Furthermore, moderate-intensive exercise (>10 h/week) stimulated coronary collateral blood flow and improved diastolic heart function in CAD patients (106). In this prospective study, 60 patients were randomly assigned to high intensity exercise, moderate intensity exercise or control for 4 weeks. Angiography identified significantly increased coronary flow index in the exercise treated groups (39 and 41%, respectively) vs. control which was associated with increased VO_2_ peak (maximal oxygen uptake; measure of aerobic fitness) (106). The authors proposed two mechanisms: recruitment of pre-existing vessels or improved EC function of small intramyocardial vessels (106). Because supervised or home exercise programs are first-line therapy for PAD (101), these mechanisms may also be relevant in limbs. Indeed, exercise training improved brachial artery dilator function in older sedentary females, (107) but these findings are not as apparent in PAD. There is also potential for long-term exercise therapy to improve systemic inflammation since an inverse correlation between exercise, inflammation and plasma CRP levels exist (108). The role of exercise treatment on inflammation in PAD and its effect on EC function is unclear and requires further elucidation.

### Cilostazol

Phosphodiesterases (PDE) play important role(s) in barrier function by inactivating the messenger cyclic nucleotides cyclic adenosine monophosphate (cAMP) and cGMP and ECs express 5 PDEs, namely, PDE1, PDE2, PDE3, PDE4, and PDE5. Cilostazol is a PDE3 inhibitor and antiplatelet medication used to relieve PAD patients from symptoms of intermittent claudication, which improves walking distance (109), in part by acting as a vasodilator and its ability to stimulate NO release. The most recent Cochrane review, which included 8 placebo-controlled randomized controlled trials involving 2,360 participants with PAD, reported that cilostazol significantly increased maximum walking distance (mean difference 39.6 m. 95% CI 21.8, 57.3; GRADE criteria very low certainty evidence) (110). However, cilostazol was associated with an increased odds of headache which is a common reason for discontinuation. It was suggested that the effects of cilostazol may vary depending on its ability to convert into its active metabolite *via* the cytochrome P450 system (111). Interestingly, cilostazol may have sex-dependent effects in ECs since female ECs express more *Pde3b* mRNA than male cells (112). However, no reports of differing responses in men and women have been identified. How cilostazol effects EC function(s) in PAD is not fully established.

### Mirabegron

Mirabegron is a second generation β_3_AR agonist used to treat overactive bladder. The STAR-PAD trial is a Phase II, multicenter, double-blind, randomized, placebo-controlled trial of mirabegron vs. placebo on walking distance in patients with PAD that is currently recruiting by Figtree and colleagues (ACTRN12619000423112) (113). A total of 120 patients aged ≥40 years with stable PAD and intermittent claudication will be randomly assigned (1:1 ratio) to receive either mirabegron (50 mg orally once a day) or matched placebo for 12 weeks. The primary endpoint is change in peak walking distance assessed by a graded treadmill test. Secondary endpoints include: (i) initial claudication distance; (ii) average daily step count and total step count and (iii) functional status and quality of life assessment. Mechanistic sub-studies will examine potential effects of mirabegron on vascular function, including brachial artery FMD, arterial stiffness and angiogenesis. Given that mirabegron is well-tolerated and clinically available for alternative purposes, a positive study is positioned to immediately impact patient care.

### Medications used for coronary microvascular dysfunction (CMD)

Large vessel blockages may not be the only mechanism contributing to PAD pathogenesis. Interestingly, microvascular dysfunction in the limb can increase amputation risk by ~20-fold, even in the absence of large vessel atherosclerosis (7). This is somewhat reminiscent of female patients with CMD who present with dysfunction of the small coronary vessels in the absence of atherosclerosis. These women have worse heart function and blood perfusion (114), with EC dysfunction the primary cause (115). Similar mechanisms may be at play with PAD, given majority of patients do not present with typical symptoms.

Central mechanisms thought to govern CMD include enhanced vasoreactivity at both epicardial and microvascular levels, impaired coronary vasodilator capacity, and increased microvascular resistance; effects of dysfunctional ECs (116). The mainstay of therapies for CMD are β-blockers, statins, calcium channel blockers and angiotensin converting enzyme (ACE)-inhibitors. These are also recommended as secondary prevention for PAD (Table 1). β-blockers and calcium channel blockers reduce severity of anginal symptoms and improve exercise stress test performance (117) and this may be due, in part, to the fact that they also block oxidative stress, improve EC survival (118), reduce EC activation and inflammation, and stimulate eNOS production (119, 120). Furthermore, β-blockers reduce FMD in people with cardiovascular diseases (121) whereas ACE-inhibitors have only showed modest improvement in FMD in patients with CAD (122), even though they modulate survival of ECs (123). Statins not only reduce cholesterol synthesis, but also dampen inflammation. They also do this *via* their direct effect on ECs. For example, low dose statins improved viability, reduced VCAM-1 and ICAM-1 expression, and atherosclerosis in pre-clinical models (124, 125). They also promoted NO release and repair mechanisms following EC injury (126). Although these medications are recommended for PAD treatment, adherence to these is variable amongst patients (127).

### Anti-inflammatory treatment

As described earlier, inflammation contributes to atherosclerosis pathophysiology, in part, *via* endothelial activation, and recruitment of leukocytes to the vessel wall. The Canakinumab Anti-inflammatory Thrombosis Outcome Study (CANTOS), a randomized double-blind, placebo-controlled trial of canakinumab, a monoclonal antibody targeting IL-1β showed that 150 mg of canakinumab reduced recurrent cardiovascular events in patients with stable CAD when compared to placebo (128). Thus, anti-inflammatories could also hold promise for PAD therapy not only in their ability to reduce inflammation, but also since many anti-inflammatories impact EC function(s). For example, anakinra, an inhibitor of IL-1, reduced endothelial dysfunction in diabetic rats; a finding that was associated with decreased NOX and circulating inflammatory cytokines including IL-1β and TNFα (129). Anakinra treatment for 30-days also improved FMD in patients with rheumatoid arthritis (130). Similar findings were also observed with TNFα inhibitors (131). More recently, colchicine has shown considerable promise as a relatively safe, inexpensive dedicated agent which targets inflammation by attenuating NLRP3 activity and IL-1β expression; a recent meta-analysis demonstrating reduced MACE, MI, stroke, and the need for coronary revascularization in patients with coronary disease (132). Data on direct effects of colchicine on ECs is limited, however an older study showed that colchicine reduced the number of E-selectin molecules on the endothelium and subsequent adhesiveness of the ECs to IL-1 or TNFα (133). Although one recent study found no difference in FMD between low-dose colchicine and placebo in patients with CAD (134), another study showed improvement in FMD in patients with a white blood cell count ≥7,500 mm^3^ (135). Never-the-less, these studies suggest that anti-inflammatory agents could be used to reduce inflammation in PAD and have potential direct effects on EC dysfunction.

### Platelet and thrombosis inhibitors to prevent PAD complications

As described earlier PAD is associated with dysregulated platelet activation and coagulation, which could precipitate major thrombotic events, observed not only in large vessel disease, but also in small vessels with microthrombi resulting in reduced tissue perfusion. Although current guidelines recommend the use of antiplatelet and antithrombotic medication to reduce the risk of MI or stroke (4, 99–101) there is a significant lack of guideline adherence (136), especially for newly-diagnosed PAD patients (137). Generally speaking, aspirin and clopidogrel are the two most studied antiplatelet medications. Aspirin inhibits COX and subsequent thromboxane A2, which is not only vasoconstrictive, but also activates platelets. Clopidogrel on the other hand prevents platelet activation by blocking the P2Y12 receptor on the surface of the platelet. Both have direct effects on ECs. For example, aspirin protected ECs against oxidized low-density lipoprotein, high glucose, angiotensin II, and H_2_O_2_-induced injury (138, 139). It also improved impaired acetylcholine-induced vasodilation in patients with atherosclerosis (140) and in pre-clinical models of aging (141). LPS-induced mRNA expression of inflammatory cytokines was attenuated with clopidogrel, associating with improved EC viability, migration, proliferation, and angiogenesis (142). Clopidogrel also prevented endothelial dysfunction in hypertensive rats (143).

Unlike anti-platelet medications, anti-coagulants inhibit the coagulation cascade and the formation of fibrin. An example is Rivaroxaban, a specific inhibitor of factor Xa. In the COMPASS trial which included >27,000 patients with stable CAD or PAD, patients were given low-dose rivaroxaban (5 mg, twice daily) and aspirin (100 mg, once daily), or aspirin alone. Patients assigned to low-dose rivaroxaban plus aspirin had better cardiovascular outcomes after ~2 years, including reduction in the combined risk of cardiovascular death, stroke, and MI (144). However, the risk of major bleeding events increased (144). In a sub-study, rivaroxaban plus aspirin reduced the incidence of major adverse limb events including amputations, when compared to aspirin alone (145). While the risk of major bleeding events was increased, the risk of fatal bleeding was not (145). In the more recent VOYAGER PAD trial (146), >6,500 PAD patients undergoing revascularization received either rivaroxaban (2.5 mg, twice daily) plus aspirin (100 mg, once daily), or aspirin alone for 3 years (147). A significant reduction in ischemic limb events including acute limb ischemia, amputation as well as cardiovascular outcomes (death, MI, stroke) were observed (147). Despite women having higher total cholesterol and greater prevalence in hypertension, diabetes and chronic kidney disease, the net clinical benefit of rivaroxaban plus aspirin was similar with sex, with comparable rates for cardiovascular outcomes and bleeding between men and women (148). The risk of major bleeding was still higher with rivaroxaban treatment.

Interestingly, rivaroxaban also has direct effects on ECs and the endothelium. Rivaroxaban administration improved vasodilation in diabetic wildtype mice, in part by increasing aortic eNOS activity (149). Forearm blood flow was also improved in diabetic patients administered rivaroxaban for 20 weeks, although treatment was associated with higher bleeding events (150). *In vitro*, rivaroxaban reduced ROS (reactive oxygen species), improved DNA repair (151) and reduced inflammatory gene expression in ECs exposed to hydroxycholesterol (152, 153). Rivaroxaban also stimulated blood flow and increased capillary density in a mouse model of diabetic PAD (154). The same group demonstrated improvement in endothelial progenitor cell migration and senescence, associating with increased eNOS activity in a hyperglycemic environment (154), suggesting that rivaroxaban has pleiotropic functions in ECs. These findings demonstrate that antiplatelet and anticoagulants may improve EC dysfunction in PAD, however additional studies are needed to fully characterize these effects.

### Glucose lowering treatments

It is well established that diabetes mellitus increases the risk of PAD and accelerates atherogenesis. Although it is unclear if intensive glucose control reduces the risk of PAD, studies describe positive outcomes in lower-extremity events including a 31% reduction in risk of amputation with intensive glucose lowering (155). As such, glucose lowering is a recommended PAD guideline pharmacotherapy; its impact on PAD has been reviewed (156). Because hyperglycemia and insulin resistance can facilitate EC dysfunction (157), many glucose-lowering therapies can impact ECs directly. Insulin for example can directly regulate eNOS expression and NO release to cause vasodilation (158), and also *via* this pathway, inhibit platelet hyperactivity (159). Further, insulin has regenerative and healing capacity in ECs by stimulating angiogenesis (160). Similarly, metformin was shown to improve endothelial-dependent vasodilation in diabetic atherosclerotic mice (161), however, its role in angiogenesis is conflicting (162, 163). More recent studies demonstrate that diabetic patients treated with glucagon-like peptide agonists, sodium-glucose co-transporter-2 inhibitors or in combination, showed improved systolic blood pressure, endothelial glycocalyx thickness and cardiac function (164). This may in part be due to the direct effect of these therapies on EC functions. For example, glucagon-like peptide 1 and sodium-glucose co-transporter-2 inhibitors reduce EC ROS production, reduce adhesion molecule expression and inflammation, improve vasodilation, and stimulate angiogenesis (165, 166). Additional studies are needed to fully comprehend the effect of glucose-lowering agents on EC functions and their cardioprotective effects.

### Emerging diagnostics and therapies

MicroRNAs (miRNAs) are small ~20–25 nucleotide long endogenous non-coding RNA sequences; their main function to regulate protein expression post-transcriptionally. miRNAs are emerging as a biomarker and potential PAD therapeutic. Using next generation genome-wide sequencing, a recent study led by Syed and colleagues identified miRNA-1827 to be significantly upregulated in the blood and plasma of patients with CLTI (167). miRNA-1827 was shown to inhibit cell proliferation and tumor angiogenesis in zebrafish (168) and may in part, contribute to impaired angiogenesis and EC function observed in PAD. miRNA-503 is also upregulated in amputated ischemic limb tissues from diabetic CLTI patients, in ischemic tissues of diabetic mice as well as in ECs exposed to diabetic conditions *in vitro* (169). Indeed, the authors found that miRNA-504 overexpression inhibited glucose-induced *in vitro* processes of angiogenesis, whereas inhibition of miRNA-503 improved blood perfusion and increased EC capillary density in diabetic mice following ischemic injury (169). These findings imply that miRNA-503 could be targeted for improving EC function in PAD. Other miRNAs are also altered in PAD patients including miRNA-130a,−27b and−210. Interestingly, miRNA-130a suppression was shown to increase angiogenesis and improve neurological function in ischemic stroke (170) and miRNA-27b inhibited human umbilical vein EC proliferation, migration and tubulogenesis by directly suppressing VEGF-C (171). In contrast, miRNA-210 stimulated pro-angiogenic processes in hypoxia in the brain and in ECs *in vitro* (172). The mRNA expression of all 3 miRNAs were increased in serum from atherosclerotic obliterans/PAD patients at I-III Fontaine stages (173), however, their role in EC functions in PAD is unclear. miRNAs could be the future in PAD diagnostics and gene therapy [reviewed in (174–176)].

In addition to miRNAs, dysfunction to the endothelium can stimulate the release of endothelial-derived microvesicles (EMVs). These are small vesicles (~0.5–2 μm) released by activated ECs during inflammation to regulate multiple cellular and vascular functions (177), playing a role in immunity, inflammation, and thrombosis (178). They can also carry miRNAs (178, 179). Because of these functions, EMVs are emerging biomarkers with therapeutic potential, particularly in atherosclerosis. For example, EMVs isolated from patients with CAD stimulated permeability and increased the mRNA expression of ICAM-1, VCAM-1, and CCl-2 in ECs *in vitro* (180). The role of EMVs in PAD is not elucidated, however, similar mechanisms may be at play. Further study is needed to understand their role in PAD.

## Future perspectives and concluding remarks

The etiology of PAD is multifactorial, and the endothelium may hold clues into pathogenesis. From its anti-inflammatory, anti-thrombotic, anti-atherogenic and pro-repair and regeneration role, the endothelium is critical in mediating cardiovascular homeostasis. Currently, there are limited treatments for limb ischemia. Alternate or novel treatments that could restore EC function(s) could have significant therapeutic implications for PAD given that EC dysfunction is a common factor facilitating pathogenesis. New therapies reducing symptoms and the risk of amputation could be life changing for these patients.

Many questions remain. For example, is the pathogenesis of PAD and other atherosclerotic disease distinct? What triggers thrombosis in PAD patients? How does the spectrum of EC phenotypes affect EC function in PAD pathogenesis? What about the potential role of antioxidants in PAD therapy or EMVs and microRNAs? What about epigenetic changes which involve miRNAs, histone modification and DNA methylation (176)? Can biomarkers such as hs-CRP, fibrinogen and D-dimer (amongst others), recently identified to predict major adverse cardiovascular outcomes in PAD (22) be used for treatment selection? For example, could PAD patients with fibrinogen levels ≥446.35 mg/L, increasing risk of cardiovascular mortality (22) be given more intensive treatment with anti-coagulants? Indeed, biomarkers could potentially be used to identify PAD patients with greater risk of adverse outcomes and those patients who may show benefit from intensive treatment (22).

Sex-dependent differences are also emerging in PAD. PAD appears to be more prevalent in women >20 years of age (181), with women more likely to be asymptomatic and have worse outcomes to treatment (182). How EC dysfunction contributes to sex-dependent differences is completely unknown and it is tantalizing to speculate that mechanisms occurring in CMD may also play a role in women with PAD. More studies are needed to identify whether EC dysfunction and the spectrum of EC phenotypes reflect sex-dependent differences in pathogenesis. Since many patients are asymptomatic or with atypical symptoms, also raises the question of identifying a non-invasive technique to measure EC functions in the clinic, particularly those that may be high risk of CLTI. FMD is used as a current strategy, however, more improved assessments with greater sensitivity and specificity are needed to take into consideration macrovascular vs. microvascular effects of ECs in PAD. Indeed, a universal method for FMD measurements and newer technologies for assessing EC functions were recently proposed in a position statement by the European Society of Cardiology Working Groups (13).

There is also a gap in knowledge in our understanding of the interaction of the endothelium with the cellular and humoral immune system in PAD, which requires further investigation. Medical therapy and secondary risk prevention for PAD described earlier include statins, antiplatelets, antihypertensives, control of diabetes, and cessation of smoking, with many of these directly affecting EC functions(s), however there is a substantial lack of guideline adherence, with only ~11–67% reported to adhere to PAD recommended guideline therapy (136). Surprisingly, PAD patients are also less likely to receive these medications than patients with CAD (127), thus, the benefit and impact of these on PAD pathogenesis including effects on EC function(s) is not fully established and requires greater study.

Finally, a greater understanding of PAD pathogenesis and mechanisms of EC dysfunction are essential. Multi-omic approaches, combining genomics, proteomics, metabolomics with phenotypic data and network biology analysis, are underway to decipher these mechanisms in PAD. Pathogenic characterization at the molecular and cellular levels could identify strategic targets leading to improvements in diagnosis, management, and treatment of PAD.

## Author contributions

MK was responsible for conception and design. MK, CB, CS, FP, SP, JL, GF, JG, SA, and DR drafted the manuscript. CS and SC designed the figures. All authors provided intellectual content and approved the manuscript for publication.

## Funding

MK and SC are supported by grants from the Australian National Health and Medical Research (NHMRC; 1188218) and the Heart Research Institute. JG is supported by grants from the NHMRC (1180736), the Medical Research Future Fund (2015979/2015817/MRAF000042) and Australian Heart Foundation (105529).

## Conflict of interest

The authors declare that the research was conducted in the absence of any commercial or financial relationships that could be construed as a potential conflict of interest.

## Publisher's note

All claims expressed in this article are solely those of the authors and do not necessarily represent those of their affiliated organizations, or those of the publisher, the editors and the reviewers. Any product that may be evaluated in this article, or claim that may be made by its manufacturer, is not guaranteed or endorsed by the publisher.
